# Effects of Steel Fibers (SF) and Ground Granulated Blast Furnace Slag (GGBS) on Recycled Aggregate Concrete

**DOI:** 10.3390/ma14247497

**Published:** 2021-12-07

**Authors:** Jawad Ahmad, Rebeca Martínez-García, Maciej Szelag, Jesús de-Prado-Gil, Riadh Marzouki, Muwaffaq Alqurashi, Enas E. Hussein

**Affiliations:** 1 Department of Civil Engineering, Military College of Engineering (Nust), Risalpur 24080, Pakistan; 2 Department of Mining Technology, Topography, and Structures, University of León, 24071 León, Spain; jesusdepradogil@gmail.com; 3 Department of Construction, Lublin University of Technology, Nadbystrzycka 40, 20-618 Lublin, Poland; maciej.szelag@pollub.pl; 4 Department of Chemistry, College of Science, King Khalid University, Abha 61413, Saudi Arabia; rmarzouki@kku.edu.sa; 5 Department of Civil Engineering, College of Engineering, Taif University, Taif 21944, Saudi Arabia; m.gourashi@tu.edu.sa; 6 National Water Research Center, P.O. Box 74, Shubra El-Kheima 13411, Egypt; enas_el-sayed@nwrc.gov.eg

**Keywords:** compressive strength, durability, sustainable concrete, acid resistance

## Abstract

Recycled aggregate is a good option to be used in concrete production as a coarse aggregate that results in environmental benefits as well as sustainable development. However, recycled aggregate causes a reduction in the mechanical and durability performance of concrete. On the other hand, the removal of industrial waste would be considerably decreased if it could be incorporated into concrete production. One of these possibilities is the substitution of the cement by slag, which enhances the concrete poor properties of recycled aggregate concrete as well as provides a decrease in cement consumption, reducing carbon dioxide production, while resolving a waste management challenge. Furthermore, steel fiber was also added to enhance the tensile capacity of recycled aggregate concrete. The main goal of this study was to investigate the characteristics of concrete using ground granulated blast-furnace slag (GGBS) as a binding material on recycled aggregate fibers reinforced concrete (RAFRC). Mechanical performance was assessed through compressive strength and split tensile strength, while durability aspects were studied through water absorption, acid resistance, and dry shrinkage. The results detected from the different experiments depict that, at an optimum dose (40% RCA, 20%GGBS, and 2.0%), compressive and split tensile strength were 39% and 120% more than the reference concrete, respectively. Furthermore, acid resistance at the optimum dose was 36% more than the reference concrete. Furthermore, decreased water absorption and dry shrinkage cracks were observed with the substitution of GGBS into RAFRC.

## 1. Introduction

Recycled concrete aggregate (RCA) is made by crushing, sieving, and cleaning this waste material from demolished concrete. Using recycled aggregates (RA) likewise decreases the raw material demand by extracting them from the ground, which also reduces the negative environmental effects. By volume, RA comprises 60–70% of natural aggregates and 30–40% mortar from old cement. Compressive strength and other characteristics of RCA are affected by parent concrete properties, workability, mix proportion, etc. [1]. The mechanical and durability performance of RCA is very low compared to concrete with natural aggregates. This lower performance of RCA could be reduced by utilizing fractional substitution of pozzolanic materials and mineral admixtures. These materials and admixtures enhance durability by filling the RCA porous microstructure and thus reducing the RCA permeability [2]. It has been concluded that RCA reduced the performance of concrete considerably. Therefore, it is recommended that it is necessary to use cementitious materials in recycled aggregate concrete to improve its permeance [3]. It has also been recommended that it is necessary to add secondary cementitious materials such as fly ash, silica fume, and GGBS into the recycled aggregate concrete to improve their performance [4].

Different cementitious materials such as fly ash [5], silica fume [6], waste glass [7], wheat straw ash [3], marble waste [8], bentonite clay [9] as well as GGBS [10] are used in concrete production. Ground granulated blast-furnace slag (GGBS) is an industrial waste by-product material that is rich in amorphous calcium, silica, and alumina, which makes it appropriate to utilize as a binder in cement concrete production [11]. GGBS is widely used in several civil engineering projects to replace cement in concrete production [12]. GGBS is an industrial by-product achieved in steel production and is generally utilized as a binding material in cement concrete production, since it enhances the mechanical performance of concrete and decreases permeability by improving the interface with the aggregate. Economic and ecological advantages in the form of power savings and source saving can also be accomplished by using GGBS as a binding material in concrete production [13]. Furthermore, the substitution of ordinary Portland cement (OPC) decreases the release of greenhouse gases and utilization of unnecessary power [14]. Mechanical performance and long-term material properties similar to those of OPC, besides its charge-effectiveness and environmental friendliness, ground granulated blast-furnace slag-based geopolymer concrete has developed an elegant solution to scholars as an alternate binding material instead of cement in concrete production [15]. Blast-furnace slag (GGBS) has been studied, where the outcomes demonstrated that there was no considerable variation in the chemical composition and particle morphology of GGBS with variations to the GGBS particle size, but also proved that the variation rate of water need and the intensity shifted rapidly with the variation of ground granulated blast-furnace slag (GGBS) particles [16]. A researcher analyzed the influence of ground nano slag (GNS) on the compressive strength, porosity, sorption, and resistance to chloride ion penetration of high strength concrete. They realized that the strength and durability of high-strength concrete were good quality when the GNS substitution rate was 10% by weight of cement. Furthermore, a lesser proportion (5%) of the ground nano slag (NGS) was not well distributed and was not sufficient to achieve more strength. A large ratio (15%) of ground nano slags (NGS) resulted in enhancing the aggregated ultrafine particle size of concrete and the improper filling of pores, resulting in more strength [17]. Experimental studies were performed on the compressive strength, split tensile strength, modulus of elasticity, chloride ion migration, and resistivity of concrete combined with ultra-fine slag powder. It was noticed that concrete mixed with ultrafine slag powder had a greater early compressive strength, less permeability, and better durability after three days of curing [18]. Some studies have shown that GGBS improved the mechanical performance of concrete in terms of compressive strength more effectively than tensile strength [10]. According to past literature, concrete with the addition of cementitious materials still need tensile reinforcement to enhance their tensile capacity [19] Fibers are one of the most prevalent methods to enhance the tensile capacity of concrete [20].

A general observation is that tiny fibers are additionally imposing in decreasing the width of plastic shrinkage cracks than thick fibers, according to past literature such as in ACI 544.5R-10 [21]. The positive response of steel fibers added in concrete depends on various aspects such as diameter, length, aspect ratio, types, cross-sectional area, concrete mix design, water–cement ratio, method of mixing, etc. [22]. For engineering purposes, particularly where high strength is required, steel fiber reinforcement is most widely used due to easy fabrication, low cost as well as high performance [23,24]. However, some studies have reported that the uneven addition of steel fiber results in negative effects on the workability of fresh concrete, leading to the poor bond of fibers with surrounding concrete, thus resulting in porous concrete, and the mechanical performance of fibers reinforced concrete decreased [25,26,27]. The important feature in material energy absorption is the method of bonding between the binder matrix and fibers [28]. Their ability to slow the propagation of cracks is known to be a highly useful feature of fibers. According to [29], the utilization of polypropylene fibers leads to a major decrease in the compressive strength of concrete, and [30] revealed that 0.15% polypropylene fibers and 0.90% steels were the best combination to obtain good performance in high-strength concrete. A study determined the effect of basalt fiber on microstructure, mechanical, and shrinkage behavior of fly ash-based geopolymer concrete [31]. They observed that basalt fiber enhanced the production rate of C–S–H, which can have a positive influence on the mechanical and durability aspects of the binder. A study revealed that polypropylene fibers have a positive effect on the flexural strength of geopolymer composites [32]. It has been also reported that glass fiber reinforced polymer (GFRP)–steel composite tubes have beneficial effects on the compression behavior of the columns [33]. A study showed that seismic resistance under the combination of axial compression and reverse lateral deformation improved with carbon fibers [34]. Furthermore, dynamic performance under impact load also showed a positive response with fibers [35]. Some studies have reported that fiber improves tensile strength more effectively than compressive strength [20,36]. According to a past study, 0.3% web reinforcement (horizontal and vertical direction) improved peak load 25% more than compared to plain concrete [37]. A study observed that 1.2% of steel fibers changed the failure mode from shear to flexure failure [38]. It has been reported that steel fibers considerably improve the flexure strength and cracking performance of concrete [39].

### Research Significance

A primary objective of this research was to focus on utilizing recycled coarse aggregate (RCA) in concrete production. A brief literature review shows that RCA decreases the performance of concrete due to its porous nature, which absorbs more water, resulting in the pore in hardened concrete. The second objective of this study was to add GGBS into recycling coarse aggregate concrete to offset its porous nature by filling voids and by the pozzolanic reaction, which enhances the mechanical performance of recycled coarse aggregate concrete. Zaid et al. showed that although pozzolanic materials improved the compressive strength of concrete, however, the concrete still had less tensile capacity, resulting in brittle failure that is unacceptable [4]. Further research was recommended in [4] where fibers must be added to GGBS recycled aggregate concrete to achieve high strength, durable, and ductile concrete. Therefore, the final objective of this study was to add steel fiber to GGBS recycled coarse aggregate concrete to obtain high-strength durable concrete. Successful utilization of these materials in concrete has multiple benefits including energy-saving, economical, solving environmental pollution as well as much as better performance compared to conventional concrete.

## 2. Experimental Program

### 2.1. Materials

#### 2.1.1. Cement

Ordinary Portland cement (OPC) type-1 as per ASTM C150 [40] was used as a binding material in this study. Its chemical and physical properties are shown in Table 1.

#### 2.1.2. Aggregate

**Figure 1:**
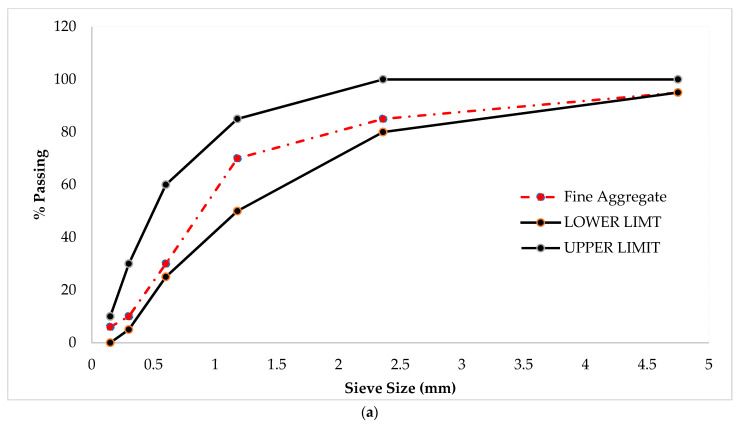

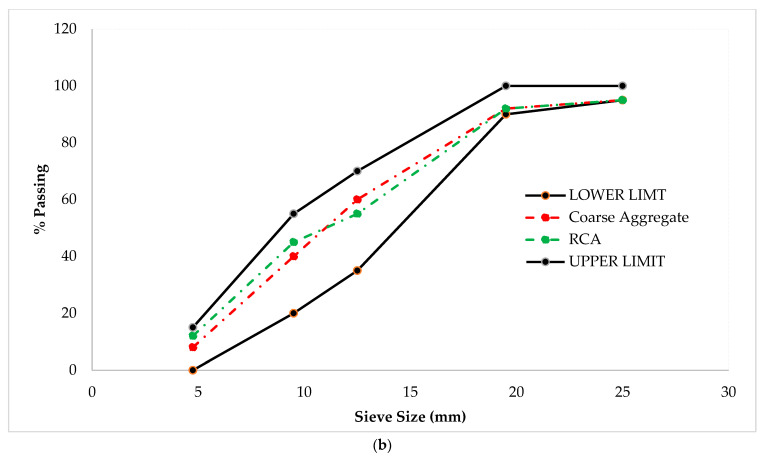
(**a**) Fine aggregate and (**b**) coarse aggregate.

Locally accessible river sand in the SSD (saturated surface dry) condition was used as a fine aggregate for all mixes. Normal weight crush stone in the SSD condition was used as the coarse aggregate for all mixes in this study, which was obtained from Margallah Wah Cantt Punjab Pakistan. Recycled aggregate was collected by crushing waste construction. Furthermore, the physical properties of the aggregates are shown in Table 2 while particle size distribution (gradation curve) was given in Figure 1.

#### 2.1.3. Ground Granulated Blast-Furnace Slag (GGBS)

**Figure 2:**
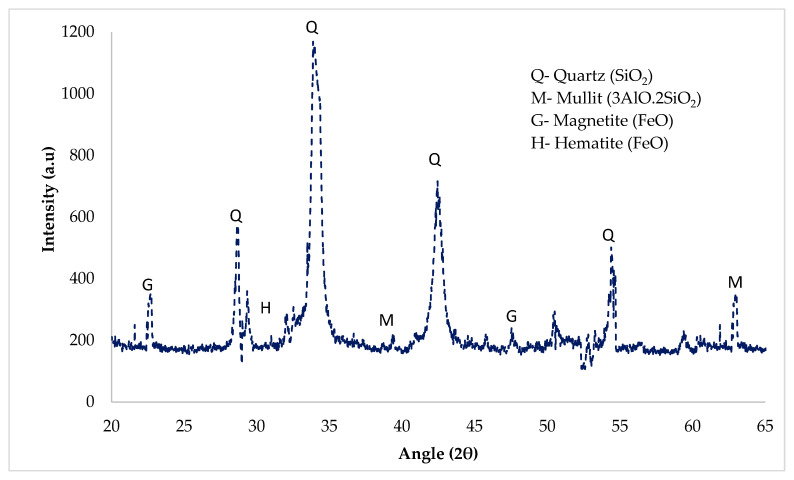
XRY pattern of GGBS.

Ground granulated blast-furnace slag (GGBS) is cheaply accessible in significant amounts and appropriate for the production of larger amounts of cement concrete production. The granulated slag is dry and ground to a fine powder, which is called ground granulated blast-furnace slag (GGBS). GGBS is usually off-white and has a bulk density of approximately 1200 kg/m^3^. The physical and chemical properties of GGBS used in this study are given in Table 3. The mineralogy of the tested samples was studied by using the results achieved through the XRD analysis. Figure 2 shows the XRD pattern of GGBS. It can be noticed that a wide range of the amorphous peak of quartz (Q) was observed at 29° and 38°, which showed the dominant amorphous nature of GGBS. Furthermore, some minor peaks of mullite (M), hematite (H), and magnetite (G) were also observed at different angles.

### 2.2. Tests Setup and Casting

The workability of fresh concrete was measured through the slump cone test in accordance with the ASTM standard [41]. Compressive strength was evaluated on a standard size cylinder 150 × 300 mm through the compressive testing machine as per ASTM standard [42]. For split tensile strength, a similar cylindrical size of 150 × 300 mm was cast and tested as per ASTM standard [43]. For the water absorption test, according to the ASTM [44], 100 mm cubes samples were cast and tested to find the water absorption of concrete. The ASTM standard [45] was used to evaluate the drying shrinkage of concrete. A 100 mm cubical sample was cast and tested to evaluate the acid resistance of concrete, which was cured of 4% sulfuric acid for a specified period. To maintain 4% concentration acid, it was changed every week. Acid attacks were calculated in terms of mass loss in percentage due to the attack of sulfuric acid. All tests were conducted after 14 days, 28 days, and 56 days of curing. Details of the mixes with different dosages of steel fibers and GGBS are provided in Table 4.

### 2.3. Mixing and Curing

Before the start of the mixing process, the necessary amount of each concrete ingredient was weighed. The speed of the mixer was kept constant at 35 rev/min for the mixing of concrete ingredients. Coarse aggregate was put into the mixture first and then fine aggregate, both materials were dry mixed, then the required amount of cement, steel fibers, GGBS were added, and superplasticizers were mixed in water, which were added over time, and mixing was conducted for about 10 min for all mixes. According to the standard ASTM [46], the casting of the sample was conducted in three different layers and each layer was compacted manually through a tamper rod with twenty-five blows. At least three samples were cast for each batch and their average value was considered as the actual result of that test. All concrete samples were cured in a temperature-controlled water tank (27 °C) until the specified time of testing. Tap water from the concrete lab was used in the tank for curing purposes.

## 3. Results Analysis

### 3.1. Fresh Properties

#### Slump and Fresh Density

**Figure 3:**
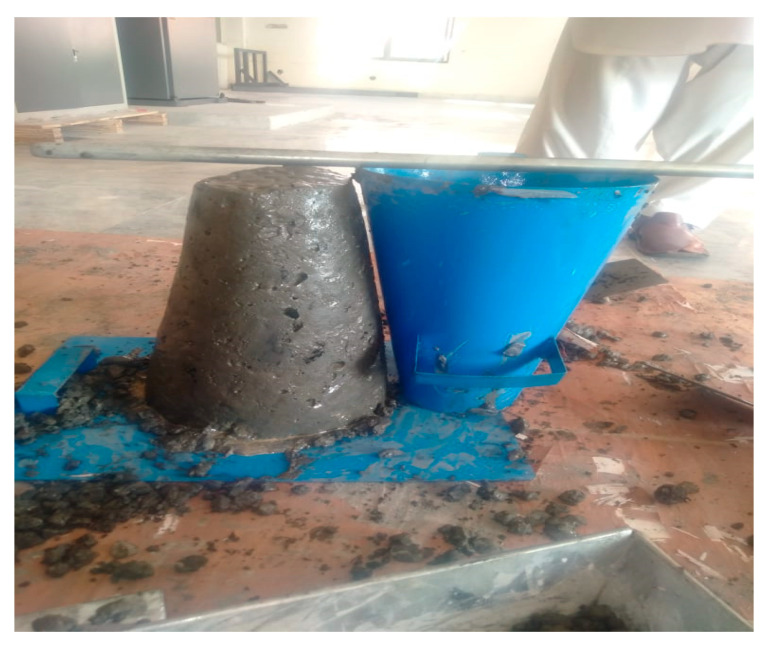
Slump test setup.

Workability of fresh concrete is a mixed property that includes the different requirements of stability, mobility, compatibility, finish ability, and place ability [47]. Figure 3 shows the slump cone test setup used in this study as per the ASTM [41].

**Figure 4:**
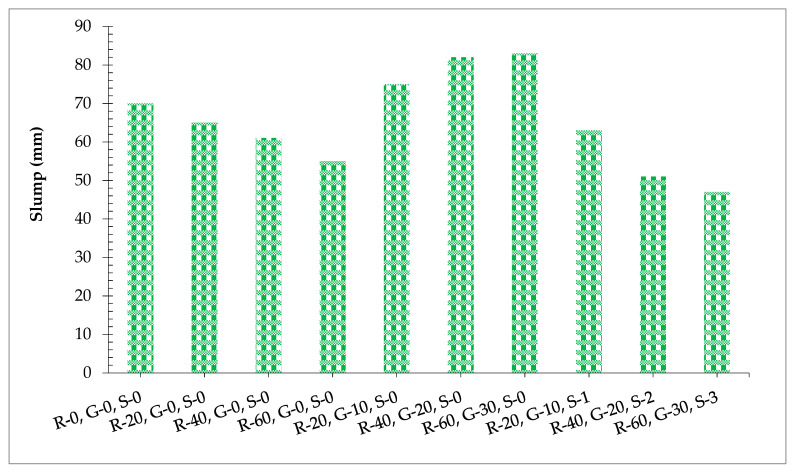
Slump results.

The workability of concrete decreased as the proportion of RCA enhanced compared to the blank mix. The maximum slump was achieved at 0% substitution of RCA while the minimum slump was obtained at 60% substitution of RCA, as shown in Figure 4. It is due to the physical features of RCA such as a porous nature that absorbs more water from the concrete mix, and hence less free water is available for lubrication to reduce the internal friction between concrete ingredients. It has been reported in the aggregate that the surface texture of RCA was rougher compared to coarse, which has an adverse effect on the workability of concrete [48]. Furthermore, when GGBS was added to the recycled aggregate concrete mix, the workability of concrete increased compared to without the GGBS recycled aggregate concrete mix. This increase in workability is due to the smooth and fine particles of GGBS [49]. The fine particles of GGBS fill in the gaps between coarse aggregate, RCA, sand, and cement, leading to more dense concrete having less voids, and hence more paste is available, which facilitates the better flow of cement concrete. It has also been reported that the greater the number of voids are present in concrete, the workability will be less as the paste fills the voids, and hence no paste is available for lubrication [3]. It is well known that many kinds of fibers reduce the workability of concrete due to the larger surface area of fibers, which increased the internal friction between the aggregate, resulting in less workable concrete [36,50]. It has also been reported that higher dosages of steel fiber require a higher dosage of superplasticizer [36]. Therefore, 1.0% of superplasticizer was kept constant throughout the studies. However, the addition of GGBS and steel fiber required a higher dosage of superplasticizer. It can be observed that the workability of recycled aggregate concrete is still improved with the incorporation of steel fiber and superplasticizer. Although it has also been reported that steel fibers decreased workability [36] while superplasticizer increased workability [51], the combined substitution ratio of steel fibers, GGBS, and superplasticizer showed better workability concrete.

**Figure 5:**
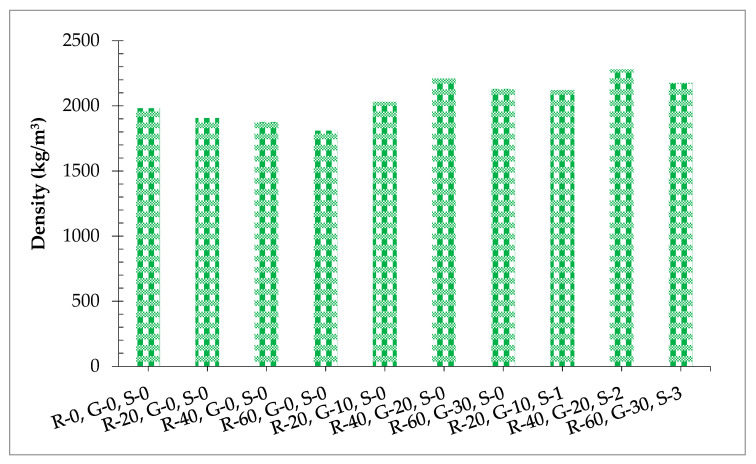
Fresh density.

Figure 5 shows the fresh density of concrete with varying dosages. Fresh density is an index that determines performance concrete (i.e., higher density results in denser concrete with less voids in hardened concrete, resulting in more strength). Fresh density decreased with the addition of RCA. Maximum fresh density was obtained at 0% substitution of RCA while minimum fresh density was obtained at 60% substitution of RCA. The negative effect of RCA could be attributed to the porous nature of RCA having large volumes with less mass, which resulted in lower fresh density compared to the control mix (concrete made with natural coarse aggregate) [48]. When GGBS was added to recycled aggregate concrete, fresh density improved as the percentage of GGBS increased. Maximum fresh density was obtained at 20% substitution of GGBS to recycled aggregate concrete in comparison to the reference mix. The positive influence of GGBS on fresh density was ascribed to the pozzolanic reaction of SiO_2_ in GGBS with calcium hydrates (CH) of cement creating secondary cementitious compounds that resulted in the greater density of concrete [49]. Additionally, GGBS fills the voids in RCA, which has porous nature, providing a more compact mass, leading to more fresh density. When steel fibers were added to GGBS recycled aggregate concrete, the fresh density of the mix still improved. It has also been reported that the fresh density of concrete decreases with the incorporation of steel fiber, particularly at higher dosage (beyond 2.0% by weight of cement) due to the lack of workability [36]. Therefore, a superplasticizer was added for each dosage of steel fiber, which results in denser concrete. When the superplasticizer was added to the concrete, it created similar charges, resulting in repelling each other and leading to more workable and dense concrete [51].

### 3.2. Mechanical Properties

#### 3.2.1. Compressive Strength

**Figure 6:**
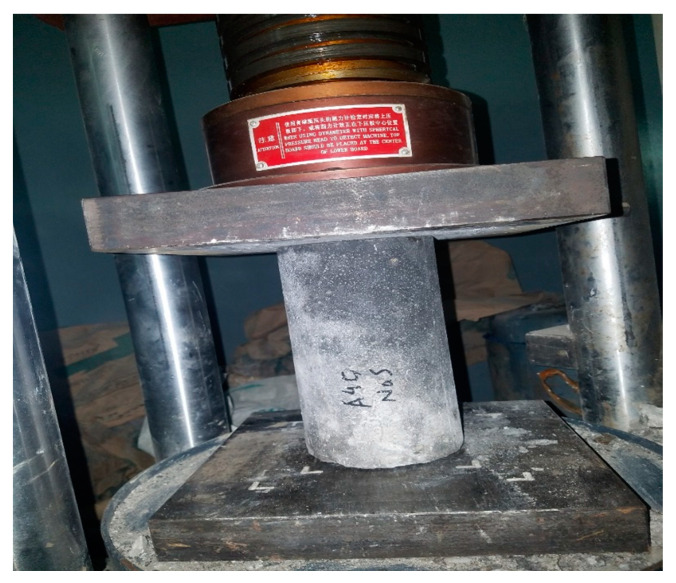
Compressive strength test setup.

The compressive strength of concrete is one of the most important properties of concrete, which can be defined as the ability of the cylindrical sample to resist stresses when it is subjected to compressive force in a compressive testing machine. A standard-sized cylinder of 300 mm in length and 150 mm in diameter was cast and tested to find the compressive strength of concrete under the ASTM standard [52], as shown in Figure 6.

**Figure 7:**
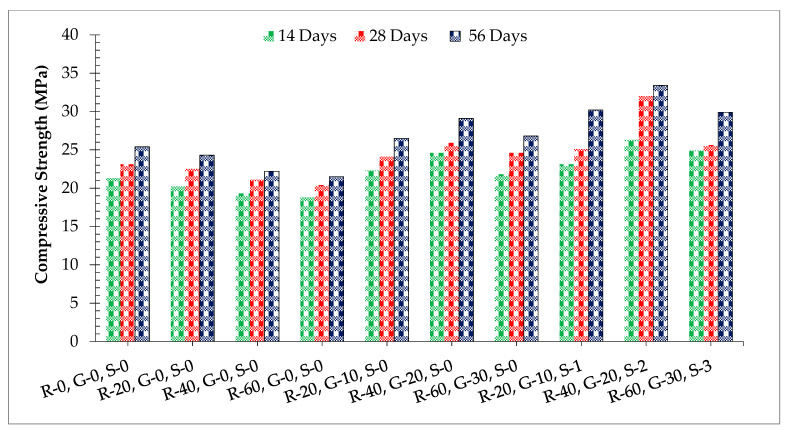
Compressive strength.

Figure 7 shows the compressive strength of different dosages of RCA, GGBS, and SF while Table 5 shows the standard deviation and coefficient of variance at different days of curing. It can be noticed that compressive strength decreases with the incorporation of RCA having a minimum strength at 60% of RCA compared to the reference concrete. This is due to the physical nature of RCA, which absorbs more water, leading to porous concrete, resulting in less compressive strength. It has also been reported that the compressive strength of recycled aggregate concrete is due to unreactive cement [4]. RCA absorbs water from concrete and no water is available for the hydration process. When GGBS was added to recycled aggregate concrete, considerable improvement in compressive strength was observed. At 28 days of curing, the compressive strength of 60% recycled aggerate concrete was approximately comparable to the control mix at 20% substitution of GGBS. The positive impact of GGBS on compressive strength was due to the pozzolanic reaction of SiO_2_ in GGBS with CH of cement, producing additional cementitious compounds [3,19]. The additional binder produced by the GGBS reaction with available lime allows concrete to continue to gain strength over time. However, at a higher dosage of GGBS (beyond 20% by weight of cement), strength reduces because of the dilution effect, which leads to the alkali-silica reaction due to a higher quantity of unreactive silica available with the high quantity of GGBS [4]. Additionally, GGBS fills the voids in the recycled aggregate, leading to denser concrete, which resulted in an improvement in the compressive strength of concrete. When steel fibers were added to GGBS recycled aggregate concrete, the compressive strength increased as the dosage of steel fibers increased up to 2.0% and then reduced. All of the fiber-reinforced concrete showed higher compressive strength compared to the control concrete with maximum compressive strength compared to the control mix at 2.0% substitution of steel fibers. The positive impact of steel fibers on compressive strength is due to the confinement of steel fibers around the cylindrical specimens. Lateral expansion is produced under the application of compressive load, which is resisted by fibers due to confinement, and as result, increases compressive strength [36].

The compressive strength of concrete improved with the addition of fibers because of the prevention of micro-cracks. When the initial cracks arrived at the fiber reinforced concrete, the path of the cracks was diverted by the matrix interface. Therefore, the concrete with fibers can endure more loads, which results in greater compressive strength. It should also be noted that the fibers have the capability of crack bridging, which acts as a crack arrestor and decreases the crack propagation in concrete. The fibers can withstand load until or unless the binder matrix pulls out [53]. This leads to the sample having high fracture energy. In addition, when the fibers are properly distributed in the matrix, then more energy is consumed to pull out to break fibers, hence leading to composites with high toughness [54].

**Figure 8:**
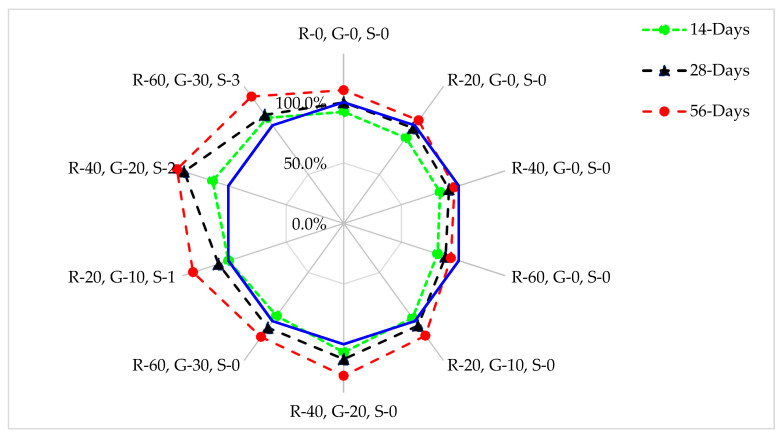
Relative analysis of compressive strength.

A similar experiment was also carried out in which 28 days of the control mix was considered the reference mix, to which the other mix was compared, as shown in Figure 8. After seven days of curing, the compressive strength was about 19% less than from the reference mix at 60% substitution of RCA. When GGBS was added to recycled coarse aggregate concrete, at 20% substitution of GGBS and 40% RCA, it showed 7.0% more than the compressive strength of the reference mix after seven days of curing. When steel fibers were added, the concrete mix of 40% RCA, 20% GGBS, and 2.0% steel fibers showed a compressive strength of more than 14% from the reference mix at seven days of curing. After 28 days of curing, compressive strength was about 13% less than from the reference mix at 60% substitution of RCA. When GGBS was added to the recycled coarse aggregate concrete, 20% substitution of GGBS and 40% RCA showed 12% more than the compressive strength from the reference mix after 28 days of curing. When steel fibers were added, the concrete mix of 40% RCA, 20% GGBS, and 2.0% steel fibers showed compressive strength greater than 39% from the reference mix at 28 days of curing. After 56 days of curing, compressive strength was about 7.0% less than from the reference mix at 60% substitution of RCA. When GGBS was added to recycled coarse aggregate concrete, 20% substitution of GGBS and 40% RCA showed 45% more than compressive strength from the reference mix after 56 days of curing. This is due to the fact that the pozzolanic reaction proceeds slowly. Some studies have also reported that early age strength was reduced with the addition of pozzolanic materials to concrete [19]. When steel fibers were added, a concrete mix of 40% RCA, 20% GGBS, and 2.0% steel fibers showed a compressive strength of more than 36% from the reference mix at 56 days of curing.

#### 3.2.2. Split Tensile Strength

**Figure 9:**
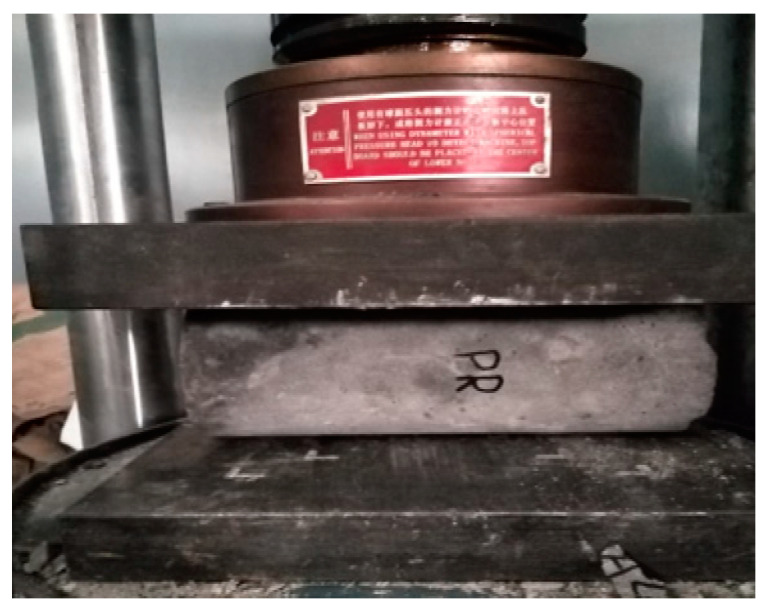
Split tensile strength test setup.

The ability of a cylindrical sample to resist the stresses when it is subject to tensile (stretching or pulling) force is called the tensile strength of that material. The direct tensile strength of concrete is not possible because of the eccentricity and grip of the sample. Therefore, indirect split tensile strength can be determined by placing the cylindrical sample in the compressive machine to split in the vertical diameter, as shown in Figure 9.

**Figure 10:**
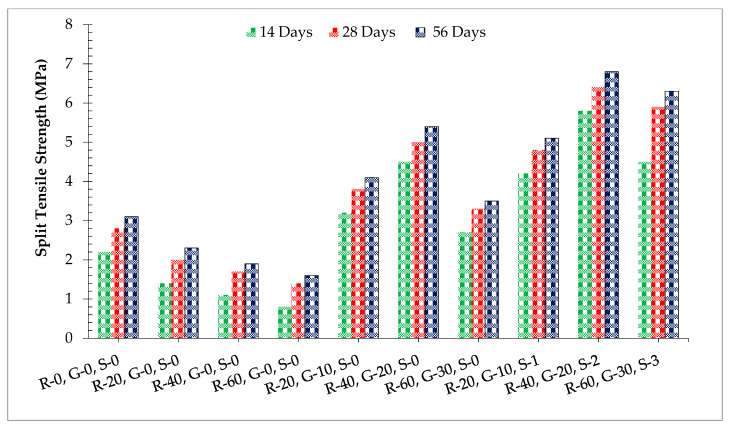
Split tensile strength.

Figure 10 shows the split tensile strength of concrete with different dosages of SF GGBS while Table 6 shows the standard deviation and coefficient of variance at different days of curing. Similar to compressive strength, split tensile strength decreased with the incorporation of RCA with a minimum strength at 60% substitution of RCA compared to the reference concrete. This is because of the physical nature of RCA, which absorbs more water, leading to pores in hardened concrete, resulting in lower split tensile strength. It is worth mentioning that split tensile more effectively decreased with the incorporation of RCA rather than compressive strength. When GGBS was added to improve the split tensile strength of recycled aggregate concrete, at 28 days of curing, the split tensile strength of aggregate 60% recycled aggerate concrete was about 10% more than the control mix at 20% substitution of GGBS. The positive impact of GGBS on split strength is because of the pozzolanic reaction of silica (SiO_2_) in GGBS with calcium hydrate (CH) of cement, giving a supplementary cementitious gel (i.e., calcium silicate hydrate (C–S–H)) [19]. According to past literature, split tensile strength mainly depends upon the binder strength. Aggregates try to move away from each other during tensile force. The additional binder produced by the GGBS offered more resistance [3]. However, at a higher proportion ratio of GGBS (beyond 20%), split tensile strength decreased because of the dilution effect, which resulted in an alkali-silica reaction. One study showed that unreactive silica (SiO_2_) is freely available at a higher dose of GGBS, which causes the alkali-silica reaction [19]. Additionally, GGBS fills the voids in the recycled aggregate, providing more compact concrete, which results in an improvement in the split tensile strength of concrete. When steel fibers were added to the GGBS recycled aggregate concrete, split tensile strength increased as the proportion of steel fibers (SFs) was enhanced up to 2.0% and then decreased gradually. All the fiber-reinforced samples showed higher strength in comparison to the reference concrete with a maximum split tensile strength at 2.0% addition of steel fibers. It is worth mentioning here that the split tensile of concrete increased more effectively than compressive strength. According to past researchers, steel fibers improved split tensile strength more effectively than compressive strength [50]. Lateral expansion is produced under the application of compressive load, which is resisted by fibers due to confinement, resulting in increased split tensile strength [36].

#### 3.2.3. Stress–Strain Curve (Uniaxial Compression)

**Figure 11:**
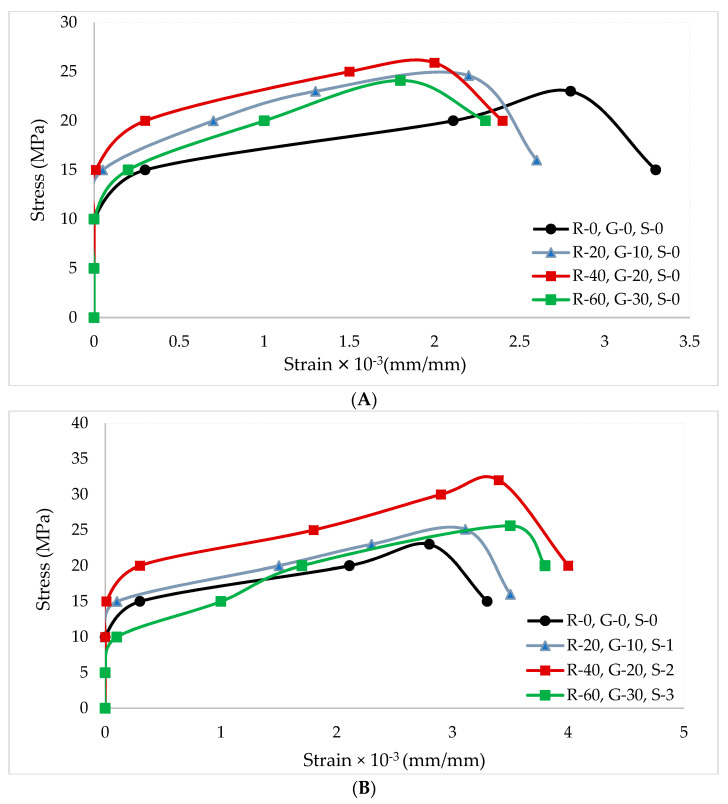
Stress–strain curve of (**A**) GGBS and (**B**) SF.

Strain gauges were attached in the direction of the compressive load. The compressive load was applied by using the compressive testing machine. P_3_ box was used to obtain strain readings. Strain readings were recorded at regular intervals. Figure 11 shows the stress–strain curve of RCA with different doses of GGBS and SF.

Stress–strain curves of both steel fiber reinforced concrete and without steel fiber concrete contained ascending and descending portions similar to the conventional concrete. From the test outcome, it can be observed that the stress required to initiate the initial strain of the steel fiber reinforced and without steel fibers reinforced concrete was approximately similar. The ultimate stress in the steel fiber reinforced concrete was greater than the concrete made with GGBS. This is due to the prevention of micro cracks. When the initial cracks arrived at the fiber reinforced concrete, the path of the cracks was diverted by the matrix interface, which resulted in more load. However, the ultimate strain of GGBS concrete was much lower than that of steel fibers reinforced concrete, which resulted in brittle failure. This is due to fact that fibers act as crack stoppers, which delay the generation of micro-cracks. It has also been reported that an increase in fiber percentages results in more ductility, toughness, and strength [55]. Adding steel fibers in concrete enhances not only the strength attributes, but also the ductility of concrete, which provides a warning (deformation) before failure [56]. Steel fiber reinforced concrete sample still carried load after the cracks appeared, which resulted in ductile failure of the concrete samples. In the descending portion of the stress-strain curve, both (steel fiber and without steel fibers) failure patterns were approximately similar.

### 3.3. Durability of Concrete

#### 3.3.1. Water Absorption

Water absorption is one of the simple tests to detect the durability of concrete. Higher water absorption results in lower durability of concrete. More water absorption also leads to freezing and thawing action, which results in the degradation of concrete. According to past literature, higher water absorption of concrete causes freezing and thawing action, particularly when concrete is placed in abruptly changing temperatures [20].

**Figure 12:**
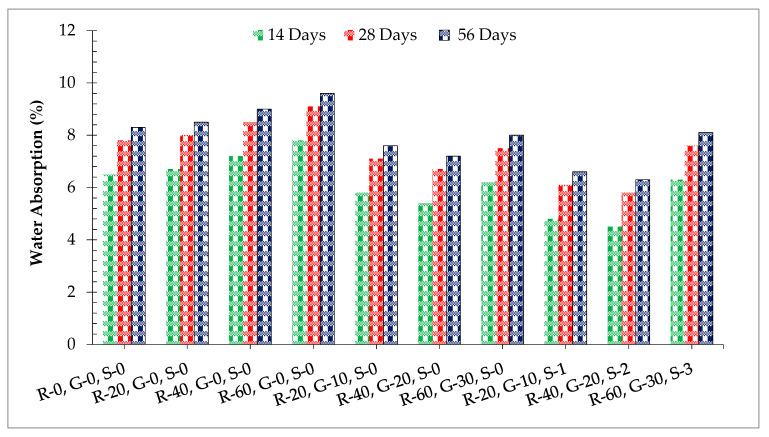
Water absorption.

Figure 12 shows the water absorption of different dosages of SF and GGBS. Water absorption increased with the incorporation of RCA having a minimum water absorption at 0% substitution of RCA compared to the control mix. RCA has a porous nature that absorbs more water from concrete, which results in more water absorption. When GGBS was added to the recycled aggregate concrete, water absorption decreased. At 28 days of curing, water absorption at 40% recycled aggerate concrete was 14% lower compared to the control mix at 20% substitution of GGBS. The positive impact of GGBS on water absorption was due to the pozzolanic reaction, which produced a more viscous binder surrounding the aggregate, which decreased water absorption [19]. Additionally, GGBS fills the void in the recycled aggregate, which results in less water absorption. However, at a higher substitution ratio of GGBS (beyond 20%), water absorption is enhanced because of the lower workability of concrete, which enhances compaction, resulting in more voids in hardened concrete [4]. When steel fibers (SFs) were added to GGBS recycled aggregate concrete, water absorption decreased as the addition rate of SF was enhanced up to 2.0% and then decreased gradually. All of the fiber-reinforced samples showed lower water absorption compared to the control having minimum water absorption after 28 days of curing at 2.0% substitution ratio of steel fibers. According to past literature, fiber protects the formation of shrinkage cracks due to which water cannot easily penetrate [20].

#### 3.3.2. Acid Resistance

Different aggressive acids are available such as HCL (hydrochloric acids), NHO_3_ (nitric acids), and H_2_SO_4_ (sulfuric acids), etc. In this study, H_2_SO_4_ (sulfuric acid) was considered as an acid attack on the concrete specimens with different proportions of RCA, GGBS, and SF.

**Figure 13:**
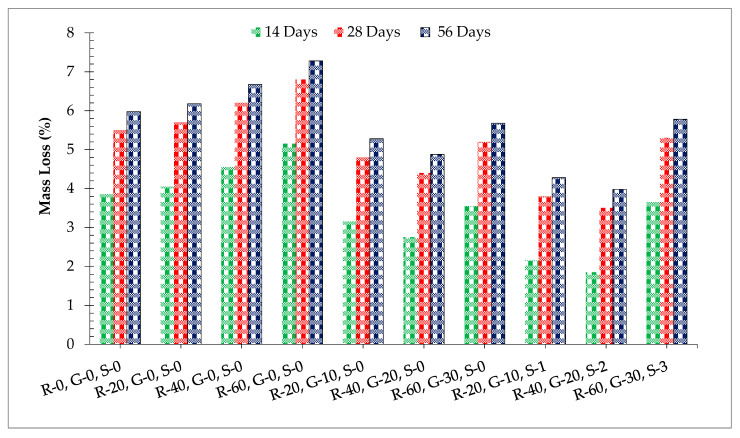
Acid resistance.

Figure 13 shows the acid resistance of different dosages of RCA, GGBS, and SF. Acid resistance increased with the substitution of RCA having a minimum acid resistance at 0% of RCA compared to the control mix. RCA has more voids than coarse aggregate through which acid can easily penetrate through concrete, leading to less acid resistance. When GGBS was added to the recycled aggregate concrete, acid resistance increased due to filling the voids and the pozzolanic reaction, which led to denser concrete through which the acid could not easily penetrate. At 28 days of curing, acid resistance of the aggregate 60% recycled aggerate concrete was 5.0% more than the control mix at 20% substitution of GGBS. However, at a higher dosage of GGBS (beyond 20% by weight of cement), acid resistance decreased due to the lower workability of concrete, which enhances compaction, resulting in more porous concrete [57]. When steel fibers were added to the GGBS recycled aggregate concrete, acid resistance increased as the dosage of steel fibers increased up to 2.0% and then reduced. All of the fiber-reinforced recycled aggregate concretes showed more acid resistance compared to the control, having a maximum acid resistance at 2.0% substitution of SF. According to past literature, fiber protects the formation of shrinkage cracks due to which acid resistance cannot easily penetrate the concrete, and hence results in more acid resistance [57].

#### 3.3.3. Drying Shrinkage

Concrete durability can also be detected through dry shrinkage tests. Water and chemicals can easily enter into the concrete body through small shrinkage cracks on the surface of the concrete, which causes the deterioration of concrete, resulting in less durable concrete.

**Figure 14:**
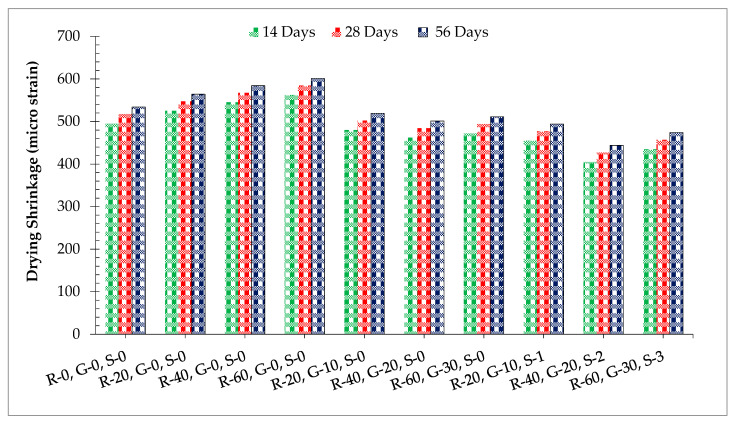
Dry shrinkage.

Drying shrinkage with respect to time for varying dosages of RCA, GGBS, and SF is given in Figure 14. It can be observed that the dry shrinkage of concrete increased as the substitution of RCA increased. It has also been reported that dry shrinkage is the movement of cement paste while the aggregate restricts this movement [54]. According to past literature, drying shrinkage is affected by the concrete porosity and stiffness [54]. RCA is basally porous, which results in more voids in hardened concrete. It has been also reported that dry shrinkage of concrete increases with the addition of RCA [4]. When GGBS is added to recycled aggregate concrete, dry shrinkage is considerably reduced. The formation of C–S–H (calcium silicate hydrate gel) due to the pozzolanic reaction causes denser concrete, which might cause a decrease in shrinkage. It has also been reported that fly ash considerably reduced drying shrinkage by filling micropores in concrete, which enhanced the internal compactness of concrete [19]. When SF is added to GGBS recycled aggerate concrete, dry shrinkage was father reduced. Furthermore, SF restricts the formation of microcracks on the surface of the concrete, which restrains the movement of harmful elements in samples, leading to minimized crack density and dimension and the elimination of the detrimental effects of drying shrinkage. The addition of SF to GGBS recycled aggregate concrete reduced the drying shrinkage due to crack prevention by SF as well as the pozzolanic reaction of GGBS. A study also showed that dry shrinkage of concrete decreased with the substitution of pozzolanic material as the pozzolanic reaction proceeds slowly compared to the hydration of cement, which results in lower heat of hydration [58]. Additionally, a study reported that the dry shrinkage crack of concrete considerably decreased with the addition of steel fibers [59].

### 3.4. Scanning Electron Microscope (SEM)

**Figure 15:**
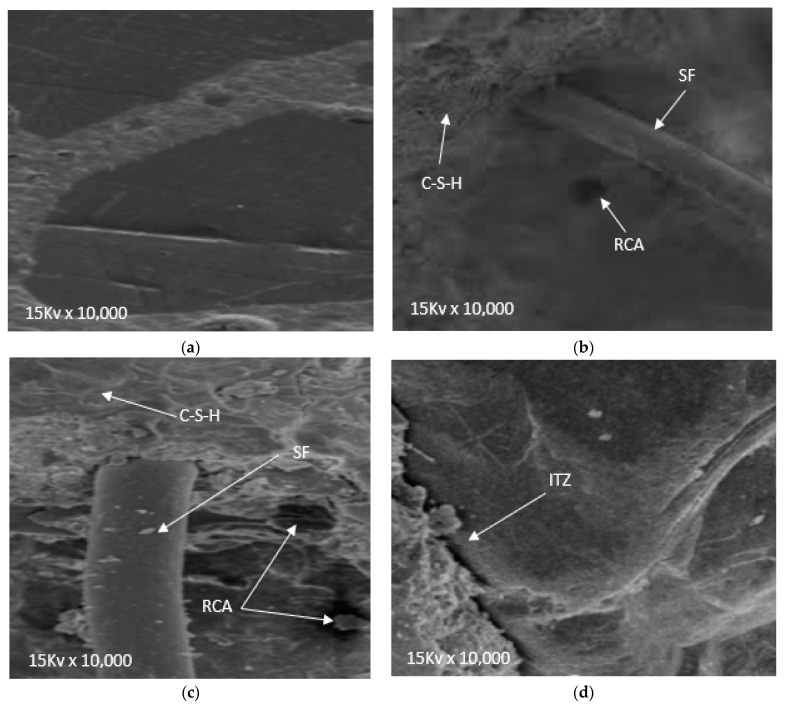
(**a**) R-0, G-0, S-0, (**b**) R-20, G-10, S-1, (**c**) R-40, G-20, S-2, and (**d**) R-60, G-30, S-3.

SEM was used to evaluate the microstructure analysis of steel fibers, GGBS, and recycled aggregate in concrete as per the ASTM C1293 267 test [60]. Scanning electron microscope (SEM) images of the concrete samples with the presence of steel fibers (SFs), GGBS, and recycled coarse aggregates in concrete after curing of 28 days are shown in Figure 15.

It is well-known that RCA decreases the mechanical and durability performance of concrete due to its porous nature, which absorbs more water from the concrete and hence no water is available for hydration and workability [54]. Therefore GGBS was added to RAC, which fills the voids in the recycled aggregate, leading to denser concrete. Due also to the pozzolanic reaction, the binding properties of cement paste are enhanced due to the formation of secondary cementitious materials (C–S–H), leading to more strength. Figure 15a–d shows the SEM test results of RAC with different doses of GGBS and SF. It can be seen that up to 40% substitution of RCA with GGBS (20%) and SF (2.0%) could improve the interfacial transition zone (ITZ) considerably. However, at 60% substitution of RCA with GGBS (30%) and SF (3.0%), a large crack (ITZ) was observed due to lack of workability, which adversely affects the durability and mechanical performance of RCA.

### 3.5. X-ray Diffraction (XRD)

**Figure 16:**
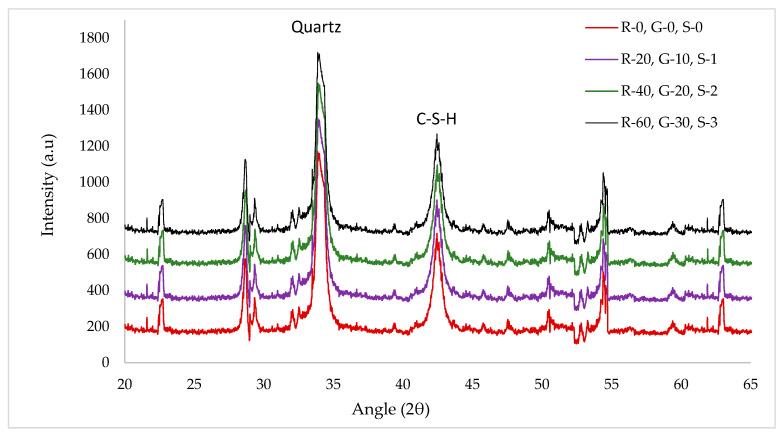
XRD pattern of varying dosages.

Figure 16 shows the XRD pattern with varying doses of GGBS to analyze the quartz and C–S–H (calcium silicate hydrate) gel in the control and GGBS substituted batches. Peaks of C–S–H gel at 30° and 45° were selected for analysis. For the control sample without GGBS, quartz was compared to C–S–H. During the hydration of cement, C–S–H is formed due to the chemical reaction quartz (SiO_2_) with lime (CH). The quantity of calcium hydrate (CH) is more than SiO_2_, which converts all SiO_2_ into the C–S–H gel and hence no further silica (SiO_2_) is available for the reaction with CH, which forms the C–S–H gel. All silica (SiO_2_) converts into C–S–H gel. CH remains unreactive, forming weak pockets resulting in lower strength of concrete. It has been also reported that CH reacts with another chemical ingredient present in concrete, resulting in less durable concrete [57]. Additionally reported is that pozzolanic materials must be added to utilize CH, which is a by-product formed during the hydration process of cement to obtain high strength durable concrete [57]. BGGS, which is rich in silica, is added to neutralize CH. It can be seen from the XRD analysis that the peak of calcium hydrate silicate (C–S–H) was increased as the percentage of GGBS increased. Maximum C–S–H peaks were observed when the substitution rate of GGBS was 30% and the minimum peak of C–S–H was observed at 0% substitution of GGBS. This is due to the pozzolanic reaction that gives the secondary (C–S–H) gel.

## 4. Conclusions

The effect of GGBS and steel fibers on machine performance, durability aspects, and microstructure analysis of recycled aggregate concrete were studied in this research. Based on experimental results, the following conclusions can be drawn.

- The workability of fresh concrete decreased as the percentage of RCA increased due to the porous nature of RCA, which absorbs more water. GGBS improves the workability of RAC by filling the voids of the recycled aggregate and hence more free water is available for workability. Additionally, the larger surface area of SF requires more water–cement pastes to coat them, which results in less workable concrete.
- At optimum dose (40% RCA, 20% GGBS, and 2.0% SF), compressive strength was 39% higher than the reference concrete at 28 days of curing while the split tensile strength was 120% more compared to the reference concrete at 28 days.
- Microstructure analyses such as XRD and SEM showed that GGBS has the creditability to be used as a pozzolanic material.
- Decreased in water absorption and shrinkage cracks were observed with the substitution of GGBS due to the combined pozzolanic and micro fillers, which gives a denser concrete. It also indicates that the acid resistance of concrete improved with the addition of GGBS.

The utilization of recycled concrete aggregate as a coarse aggregate in concrete protects granite stone, which is one of the quickly diminishing raw materials. When GGBS is added to concrete, the reduction in limestone, which is a raw ingredient essential for cement production, is reduced, and hence the sustainability quality in the production of concrete is enhanced. This will considerably lower the emissions of carbon dioxide, nitrogen dioxide, and other dangerous gases to the environment and assist in limestone conservation. It can be noted that the addition of steel fibers improves the concrete mechanical and durability performance with the composite addition of GGBS and recycled aggregate. Therefore, concrete production can be made sustainable by implementing the procedure used in the current study by the incorporation of recycled aggregate, GGBS, and steel fibers.

## Figures and Tables

**Table 1:** Physical and chemical properties of OPC.

Chemical Property	Percentage (%)	Physical Property	Results
CaO	62.7	Particle Size	≤75 µ
SiO_2_	22.9	Fineness	94%
Al_2_O_3_	6.4	Normal Consistency	28%
Fe_2_O_3_	2.7	Initial Setting Time	36 min
MgO	2.5	Final Setting Time	418 min
SO_3_	1.4	Specific surface	322 m^2^/kg
K_2_O	1.2	Soundness	1.60%
Na_2_O	0.2	compressive Strength	42 Mpa

**Table 2:** Properties of aggregate.

Physical Property	Fine Aggregate	Coarse Aggregate	RCA
Particle Size (mm)	4.7 to 0.075	25 to 4.75	25 to 4.75
Fineness Modulus	2.55	4.23	4.12
Absorption Capacity (%)	3.9	2.9	4.4
Moisture Content (%)	1.6	1.4	1.8
Bulk density (kg/m^3^)	1556	1580	1485

**Table 3:** Physical and chemical properties of GGBS.

Chemical Property	Percentage (%)	Physical Property	Results
Ca0	53.55	Particle Size	≤75 µ
SiO_2_	9.13	Color	White
Al_2_O_3_	20.2	Specific Gravity	2.20
Fe_2_O_3_	7.23	Type	F
MgO	4.32	Clay (%)	0.9
SO_3_	2.07	Bulk density (kg/m^3^)	1180
K_2_O	1.9		
Na_2_O	1.6		

**Table 4:** Quantification of materials per m^3^.

Mix ID	Cement (kg)	F.A (kg)	C.A (kg)	RCA (kg)	HRWR (kg)	GGBS (kg)	SF (kg)
R-0, G-0, S-0	425	625	1275	-	4.25	-	-
R-20, G-0, S-0	425	625	1020	255	4.25	-	-
R-40, G-0, S-0	425	625	765	510	4.25	-	-
R-60, G-0, S-0	425	625	510	765	4.25	-	-
R-20, G-10, S-0	382.5	625	1020	255	4.25	42.5	-
R-40, G-20, S-0	340	625	765	510	4.25	85	-
R-60, G-30, S-0	297.5	625	510	765	4.25	127.5	-
R-20, G-10, S-1	382.5	625	1020	255	4.25	42.5	4.25
R-40, G-20, S-2	340	625	765	510	4.25	85	8.85
R-60, G-30, S-3	297.5	625	510	765	4.25	127.5	12.75

R = RCA, G = GGBS, S = SF. F.A = Fine aggregate, C.A = Coarse aggregate, RCA, Recycle coarse aggregate. HRWR = High range water reducing admixture. GGBS = Ground granulated blast-furnace slag, SF = Steel fibers.

**Table 5:** Standard deviation and coefficient of variance of compressive strength (MPa).

Mix ID	14 Days		28 Days		56 Days	
	Standard Deviation	Coefficient of Variance	Standard Deviation	Coefficient of Variance	Standard Deviation	Coefficient of Variance
R-0, G-0, S-0	0.91	4.30	0.25	1.08	0.25	0.10
R-20, G-0, S-0	0.76	3.76	0.40	1.79	0.36	1.03
R-40, G-0, S-0	0.45	2.43	0.26	1.24	0.25	1.12
R-60, G-0, S-0	0.51	2.77	0.49	2.42	0.32	1.49
R-20, G-10, S-0	1.45	6.71	1.21	5.22	0.41	1.50
R-40, G-20, S-0	0.43	1.79	0.49	1.91	0.35	1.36
R-60, G-30, S-0	0.50	2.30	0.20	0.81	0.37	1.41
R-20, G-10, S-1	0.45	1.92	0.21	0.83	1.15	3.84
R-40, G-20, S-2	0.50	1.91	0.80	2.47	0.26	0.80
R-60, G-30, S-3	0.56	2.29	0.56	2.23	1.06	3.56

**Table 6:** Standard deviation and coefficient of variance of split tensile strength (MPa).

Mix ID	14 Days		28 Days		56 Days	
	Standard Deviation	Coefficient of Variance	Standard Deviation	Coefficient of Variance	Standard Deviation	Coefficient of Variance
R-0, G-0, S-0	0.23	11.14	0.25	8.88	0.37	12.35
R-20, G-0, S-0	0.10	6.66	0.20	10.07	0.20	8.92
R-40, G-0, S-0	0.21	17.88	0.15	9.16	0.30	15.78
R-60, G-0, S-0	0.15	17.62	0.10	6.67	0.20	14.52
R-20, G-10, S-0	0.32	9.84	0.20	5.67	0.21	5.11
R-40, G-20, S-0	0.36	8.01	0.47	9.51	0.30	5.80
R-60, G-30, S-0	0.25	9.43	0.37	11.95	0.15	4.44
R-20, G-10, S-1	0.15	3.58	0.47	9.91	0.15	2.90
R-40, G-20, S-2	0.36	6.11	0.40	6.28	0.32	4.84
R-60, G-30, S-3	0.30	6.83	0.40	6.69	0.26	4.19

## Data Availability

All the required data that support the finding are presented in the manuscript.
